# Root Translucency for Age-at-Death Estimation in Adults: A Systematic Review

**DOI:** 10.3390/diagnostics16183055

**Published:** 2026-09-20

**Authors:** Ana Belén Márquez-Ruiz, Lissett Ramírez-Almenares, Aurora Valenzuela

**Affiliations:** Department of Forensic Medicine, Faculty of Medicine, University of Granada, Avenida Doctor Jesús Candel Fábregas 11, 18016 Granada, Spain

**Keywords:** root translucency, age estimation, age-at-death, forensic odontology, systematic review

## Abstract

**Background/Objectives**: The estimation of age in skeletal remains is particularly challenging in adults due to the reduction in reliable morphological indicators with age. One valuable marker for this purpose is root translucency, either alone or in combination with other parameters. The aim of this systematic review was to evaluate the accuracy of root translucency as a marker for estimating age-at-death in adults while assessing its reliability, limitations, and practical utility in forensic scenarios. **Methods**: A systematic review of the literature was conducted by synthesizing the scientific evidence available in PubMed, Web of Science, Scopus, SciELO, and Google Scholar. **Results**: Thirteen articles published between 2015 and 2025 in indexed journals were selected. The evaluated methods were more accurate in middle-aged groups (mean errors lower than 10 years), with a tendency to overestimate age in young adults and underestimate it in older individuals. The impact of the trajectory effect (i.e., that estimation errors increase with age) could be reduced by employing Bayesian statistics in the analyses. Similarly, logarithmic regression offered better results in adults over 65 years of age. International databases appeared to be a promising alternative when population-specific information is unavailable, even when root translucency was the only age marker used. **Conclusions**: These new approaches, which increase the accuracy of the methods, need to be validated on larger and more diverse samples to determine their applicability to various forensic contexts and their ability to achieve acceptable results.

## 1. Introduction

Age-at-death estimation is a key aspect of anthropological and forensic research, as it helps to establish the biological profile of unidentified human remains [1]. In children and adolescents, age can be determined with reasonable accuracy due to well-defined and chronologically ordered growth and development processes. However, in adults, the degenerative changes experienced by teeth and bones are more variable [2].

Among the degenerative changes that teeth undergo with age, root translucency stands out as a reliable indicator of age [3,4]. This physiological process is due to the precipitation of hydroxyapatite crystals within dentinal tubules, which alters the refractive index of the root dentin and causes its translucency [4,5]. Root translucency becomes visible in the third decade of life at the apical end of the root and gradually progresses in a coronal direction [6]. After death, dentin translucency may decrease or be lost due to postmortem taphonomic effects [6,7].

In 1950, Gustafson [8] developed the first method for estimating age-at-death based on an analysis of root dentin translucency alongside other dental age indicators (i.e., attrition, periodontal recession, secondary dentin, cementum apposition, and root resorption). However, not all of the parameters are influenced equally by age, with dental translucency the least affected by external factors [9]. Thus, in 1970, Bang and Ramm [10] presented a more detailed method for estimating age-at-death in adults based solely on the measurement of root dentin translucency. In 1992, Lamendin et al. [3] proposed a procedure for estimating age in adults designed for practical forensic use. This procedure involves the measurement of root height, periodontal recession, and root translucency, avoiding destructive techniques.

Since then, numerous studies have tested the Lamendin method on individuals from different geographical locations [4]. In 2002, Prince and Ubelaker [11] evaluated the effects of sex and population ancestry on age estimation, creating specific regression formulas for males and females of European and African ancestry. The formulas developed by Lamendin et al. [3] and Prince and Ubelaker [11] are now the most widely employed approaches due to their simplicity, efficacy, repeatability, and reproducibility [4]. Recent research has focused on developing procedures that improve global approximations and estimates for individuals over 70 years of age [4]. Digital image analysis and computer-assisted measurements have been proposed as alternatives to conventional approaches, allowing more objective quantification of translucency-related parameters [12]. Furthermore, the introduction of the Forensic International Dental Database (FIDB) [13] represented a promising advance by incorporating Bayesian modeling into adult age-at-death estimation using root dentin translucency. Subsequent updates of the FIDB [14,15] have further refined this methodology through the development of new models and the incorporation of logarithmic transformations to improve age estimation performance.

Despite the frequency with which root translucency has been assessed in forensic contexts for age estimation, the methods for measuring it, the accuracy of the estimates, and the influence of variables such as tooth type, sex, and population ancestry vary among studies. In this context, the aim of the present systematic review was to assess whether root translucency is an accurate marker for estimating age-at-death in adults by synthesizing the available scientific evidence and evaluating its reliability, limitations, and practical utility in the forensic field. This review focuses specifically on studies published between 2015 and 2025, aiming to provide an updated overview of methodological developments in age estimation based on root dentin translucency. In addition, unlike a previous systematic review that addressed both root dentin translucency and cementum annulations [6], the present review is exclusively concerned with root dentin translucency assessed in teeth obtained from human cadavers, thereby enabling a more specific evaluation of its applicability to age-at-death estimation.

## 2. Methods

The review question was formulated using the PECO tool (Population, Exposure, Comparison, and Outcome): “In adults, is root dentin translucency an accurate marker for estimating age-at-death?” The systematic review protocol was designed according to the Preferred Reporting Items for Systematic Reviews and Meta-Analyses (PRISMA) statement [16] (Supplementary File S1) and subsequently registered in the International Prospective Register of Systematic Reviews (PROSPERO) (ID number: CRD420251022725).

A literature search was independently conducted by two reviewers (A.B.M.-R. and L.R.-A.) between April and May 2025 in four databases: PubMed, Scopus, Web of Science, and SciELO. Google Scholar was also used to find relevant studies that might not be included in the primary search sources. Various search strategies were used, employing both broad and method-specific search terms, namely, root translucency OR root transparency AND age estimation; Lamendin method AND age-at-death; and Prince and Ubelaker method AND age-at-death. For Google Scholar, the search strategies were adapted to improve search specificity using the advanced search options of the platform. Specifically, the first search strategy included at least one of the terms “root,” “translucency,” or “transparency,” together with the exact phrase “age estimation,” restricting the search to the title field. The second and third search strategies used the words “age,” “at,” and “death” (all words), combined with the exact phrases “Prince and Ubelaker method” and “Lamendin method,” respectively, allowing the search terms to appear anywhere in the article. To capture the most recent evidence, studies had to have been published within the last 10 years (2015–2025). The complete search strategies for all databases are presented in Supplementary File S2.

All retrieved references were imported into Mendeley (version 2.129.0; Elsevier, Amsterdam, the Netherlands), where duplicates were removed. Articles were independently selected by the two reviewers based on title and abstract screening (in the first stage) and full-text assessment (in the second stage) to answer the review question and determine if the articles met the previously established inclusion criteria. The inclusion criteria comprised observational, cross-sectional studies investigating age-at-death estimation methods based on root dentin translucency in adult corpses. The restriction to postmortem samples was established a priori to ensure a homogeneous evidence base and to allow the findings to be interpreted specifically in the context of forensic age-at-death estimation. We acknowledge that studies based on living subjects provide relevant evidence on the use of root dentin translucency for age estimation. However, including both living and postmortem samples would have broadened the review question and introduced additional heterogeneity beyond the specific objective of the present review. The exclusion criteria were systematic reviews, meta-analyses, case series, case reports, book chapters, studies involving teeth extracted from living subjects, and animal studies. Studies published in languages other than English or Spanish or lacking full-text availability were excluded. Any disagreements between reviewers were resolved through discussion. If consensus could not be reached, a third reviewer (A.V.) made the final decision.

The risk of bias of the selected studies was independently assessed by two reviewers (A.B.M.-R. and L.R.-A.) using the Joanna Briggs Institute (JBI) Critical Appraisal Checklist for Analytical Cross Sectional Studies [17]. This checklist consists of 8 questions evaluating methodological aspects (i.e., clearly defined inclusion criteria; a detailed description of the study subjects and setting; valid and reliable measurement of the exposure; the use of objective, standard criteria for measurement of the condition; identification of confounding factors; statement of strategies to deal with confounding factors; valid and reliable measurement of outcomes; and the use of appropriate statistical analysis) that must be answered using one of four response options: “yes,” “no,” “unclear,” or “not applicable.” Any discrepancies were resolved through discussion or with the participation of a third reviewer (A.V.), if needed. Risk of bias was considered low if the number of positive responses exceeded 70%, moderate if the number of positive responses reached 50–69%, and high if the number of positive responses did not exceed 49%. Although the JBI tools do not provide validated cut-off values for an overall risk-of-bias classification, percentage scores were calculated as a descriptive measure to facilitate the interpretation and comparison of the methodological quality of the studies included in the review, following the approach described in previous research [6].

Data extraction was performed independently by two reviewers (A.B.M.-R. and L.R.-A.). The information obtained from each study was organized into a table for qualitative analysis. The following data were collected: article identification (title, author(s), and publication year), sample size and sex distribution, age range, method used to estimate age, and the most relevant results according to the review question and specific objectives. As before, any discrepancies or uncertainties were resolved through discussion, consensus, or, if necessary, by involving a third reviewer (A.V.). Due to the substantial heterogeneity among the included studies, a meta-analysis was not considered appropriate. Therefore, a qualitative synthesis of the findings was performed.

## 3. Results

**Figure 1:**
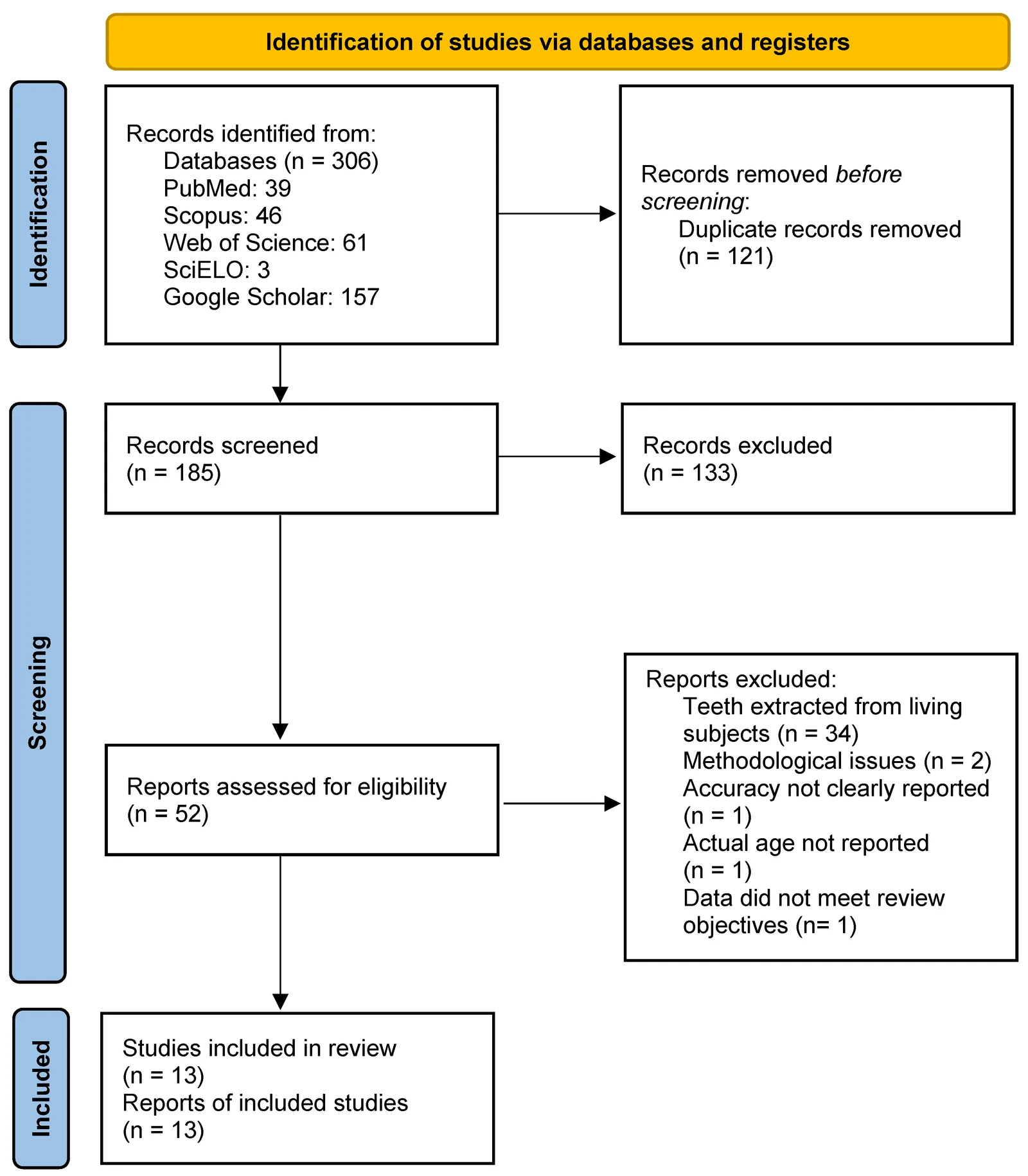
Flow diagram of the literature search and screening process according to PRISMA 2020 guidelines [16].

The searches retrieved 306 articles after excluding duplicate records within each individual database: PubMed (39 articles), Scopus (46), Web of Science (61), SciELO (3), and Google Scholar (157). Duplicate records identified across the different databases were subsequently removed, leaving 185 studies for title and abstract screening. All articles that appeared to answer the review question and met the inclusion criteria (52 studies) were selected for full-text review. Of these, 39 studies were excluded for one of the following reasons: (i) the use of teeth extracted from living subjects (*n* = 34); (ii) methodological limitations (*n* = 2); (iii) unclear or inadequately reported measurement accuracy (*n* = 1); (iv) unreported actual age of individuals (*n* = 1); and (v) data irrelevant to the review objectives (*n* = 1). Finally, 13 studies were included in the systematic review for qualitative analysis [14,15,18,19,20,21,22,23,24,25,26,27,28]. The PRISMA [16] flowchart of the literature search and screening process is presented in Figure 1.

Of the 13 included studies, 11 [14,15,18,19,20,21,22,23,24,25,26] were considered to have a low risk of bias, while 2 [27,28] were assessed to have a high risk of bias. The results of the risk of bias assessment according to the JBI Checklist for Analytical Cross Sectional Studies [17] are detailed in Table 1.

Table 2 shows the studies included in the review and a summary of the data extracted for qualitative analysis. The results of each individual study are subsequently presented, organized according to the methodological approach used for age-at-death estimation.

**Table 1:** Critical appraisal of methodological quality and risk of bias.

Authors (Year)	Inclusion Criteria	Study Setting	Exposure Measured	Condition Measurement	Confounding Factors Identified	Confounding Factors Addressed	Outcome Measure	Statistical Analysis	Percentage of Positive Answers/Risk of Bias
Alvarado & Requena (2023) [18]	Y	U	Y	Y	Y	Y	U	Y	75%/Low
Baz et al. (2022) [19]	Y	U	Y	Y	Y	Y	Y	Y	87.5%/Low
Becerra & Cortés (2020) [27]	N	U	Y	Y	N	N	U	Y	37.5%/High
De Angelis et al. (2015) [20]	Y	Y	Y	Y	Y	Y	Y	U	87.5%/Low
Garizoain et al. (2020) [21]	Y	Y	Y	Y	Y	Y	Y	Y	100%/Low
Garizoain et al. (2022) [22]	N	U	Y	Y	Y	Y	Y	Y	75%/Low
Garizoain et al. (2023) [14]	Y	Y	Y	Y	Y	Y	U	Y	87.5%/Low
Parra et al. (2025) [15]	Y	Y	Y	Y	Y	Y	Y	Y	100%/Low
Peralta et al. (2022) [23]	Y	Y	Y	Y	Y	Y	Y	Y	100%/Low
Quispe (2021) [24]	N	Y	Y	Y	Y	Y	Y	Y	87.5%/Low
Regalado et al. (2017) [28]	N	U	Y	Y	N	N	U	U	25%/High
Rocha et al. (2025) [25]	Y	Y	Y	Y	Y	Y	Y	Y	100%/Low
Zorba et al. (2018) [26]	Y	Y	Y	Y	Y	Y	Y	Y	100%/Low

Y, yes; N, no; U, unclear.

**Table 2:** Characteristics of the studies included in the systematic review.

Authors (Year)	Age Estimation Method	Sample				Conclusions
		*n* (Teeth/Individuals)	M	F	Age	
Alvarado & Requena (2023) [18]	Bang & Ramm	248/124	172 ^a^	76 ^a^	30–70	The Peruvian formula was more accurate than the original formula. Age was overestimated by both formulas in individuals < 60 years of age.
Baz et al. (2022) [19]	Lamendin	74/50	48 ^a^	26 ^a^	25–89	The Brazilian equation significantly improved the method’s performance between ages 60 and 89.
Becerra & Cortés (2020) [27]	Lamendin and supervised machine learning techniques	48/45	NR	NR	19–81	Gaussian Process Regression models presented lower errors in the estimates than those shown by traditional techniques.
De Angelis et al. (2015) [20]	Lamendin and Prince & Ubelaker	91/21	7	14	41–96	Burial, even just for a few years, can affect the accuracy of the Lamendin method.
Garizoain et al. (2020) [21]	Lamendin	457/91	57	34	18–91	Sex did not influence age estimation. The method showed better results in individuals aged 36–50 years, overestimating age in young adults and underestimating it in older adults.
Garizoain et al. (2022) [22]	Modified Lamendin (root translucency alone)	101/101	NR	NR	23–90	The logarithmic model showed lower errors in the estimates for adults over 65 years of age.
Garizoain et al. (2023) [14]	Modified Lamendin (FIDBv2)	212/212	145	67	20–>80	The most accurate estimates were obtained in individuals between 30 and 60 years of age, with errors < 10 years. FIDBv2 showed adequate performance for age estimation in individuals of different geographical origins.
Parra et al. (2025) [15]	Modified Lamendin (root translucency alone, FIDBv3)	142/142	94	48	30–80	Accurate age estimation was achieved using a Bayesian model with logarithmic transformation of the variables. The lowest errors in the estimates were reported for the 40–59 age group.
Peralta et al. (2022) [23]	Prince & Ubelaker, Ubelaker & Parra, and Vilcapoma	90/90	90	-	30–59	The Vilcapoma method showed the highest accuracy and precision for age estimation in the evaluated Peruvian sample.
Quispe (2021) [24]	Ubelaker & Parra	43/29	25	4	35–73	The method was not reliable in individuals over 50 years of age.
Regalado et al. (2017) [28]	Bang & Ramm	43/43	19	24	19–79	The method tends to overestimate actual age.
Rocha et al. (2025) [25]	Lamendin, Prince & Ubelaker, Fialho, and modified Fialho	166/166	112	54	30–86	The modified Fialho method, which focuses solely on root translucency, showed the highest accuracy for age estimation in the Portuguese sample.
Zorba et al. (2018) [26]	Lamendin and Prince & Ubelaker	1436/306	161	145	20–97	Both methods can be considered accurate for estimating age-at-death in middle-aged individuals.

*n*, number of cases; M, males; F, females; NR, not reported. ^a^ With regard to the number of teeth (not individuals).

Regalado et al. [28] measured the length of root dentin translucency in teeth recovered from a Mexican cemetery to estimate age using the method proposed by Bang and Ramm [10]. The mean difference between actual and estimated ages was −3.06 years, indicating that the method tends to overestimate actual age. The standard error of the estimates was 9.02 years. However, this study exhibited methodological weaknesses, including a lack of information or uncertainties regarding the inclusion criteria, description of the setting, handling of confounders, outcome measures, or analytical adequacy (Table 1).

Similarly, Alvarado and Requena [18], applying the Bang and Ramm method [10], developed a specific formula for the Peruvian population based on an evaluation of the percentage length of root dentin translucency. The mean difference between actual and estimated ages indicated an overestimation of chronological age for both the Bang and Ramm formula (−6.701 years) and the Peruvian formula (−5.999 years). These differences were significant for individuals aged 30 to 50 years, with an overestimation of age by around 10 years. The overestimation decreased in the 51–60 age range (Bang and Ramm formula, −3.168 years; Peruvian formula, −1.395 years) and shifted to an underestimation in older adults (Bang and Ramm formula, 1.519 years; Peruvian formula, 2.088 years). The accuracy of the new Peruvian formula was considered acceptable, with a lower mean absolute error compared to the original formula (7.83 vs. 8.97 years).

In their study, Garizoain et al. [21] assessed the effectiveness of the Lamendin method [3] in a sample of teeth from an Argentine osteological collection. The results indicated that the accuracy of this method depends on the type of tooth and the age of the individual, with age having a greater impact on the estimate. Sex did not significantly affect the results. No significant differences were observed between actual and estimated ages in cadavers aged 36 to 50 years (mean absolute error of 5 years), but there was a tendency to overestimate age in young adults and underestimate it in older individuals.

Baz et al. [19] also evaluated the usefulness and accuracy of the Lamendin method [3], in this case using a Brazilian osteological sample. They applied an equation specifically adapted to this population and compared these results with those obtained using Lamendin’s formula. The original formula yielded a general underestimation of age of 11.32 years, with overestimations of around 10 years in individuals aged 25–39 years and underestimations of around 5 years in adults aged 40–59 years. The development of a specific formula for the population clearly improved the performance of the method, resulting in smaller differences between actual and estimated ages in adults aged 60 to 89 years.

Garizoain et al. [22], in turn, developed two age-estimation models, one linear and one logarithmic, based on root translucency measurements obtained using the Lamendin method [3]. The study was conducted using a sample from an Argentine osteological collection to generate the models and another from a Spanish collection to evaluate the models generated. Age was found to influence the accuracy of the estimates, with the error increasing in older individuals and a tendency to underestimate chronological age. Linear regressions were more effective in predicting age in young adults (35–50 years), while the logarithmic model was more appropriate in adults over 65 years of age, with mean absolute errors of less than 10 years in the group aged 66 to 80 years.

Subsequently, Garizoain et al. [14] updated and extended the FIDB [13], a Bayesian age-at-death estimation model based on the criteria of the Lamendin method [3]. This new version (FIDBv2) incorporates a logarithmic transformation of the variables and additional information for calculating probabilities based on a larger number of individuals from different countries. FIDBv2 was applied to two control population samples, one from Colombia (*n* = 112) and another from Greece (*n* = 100), to evaluate the accuracy and precision levels of the method. The results of this improved version were considered satisfactory, suggesting that the method is appropriate for estimating age-at-death in adult individuals from different forensic contexts, particularly when data on their geographical origin are not available. The most accurate estimates of chronological age were achieved in individuals between 30 and 60 years of age, with mean absolute errors of less than 10 years. In individuals between 60 and 80 years old, the use of the FIDBv2 algorithm improved the estimation performance compared to previous methods. The estimates tended to overestimate age in young adults (<40 years) while underestimating it in older age groups.

Parra et al. [15] also revisited the FIDB [13], which had shown satisfactory results for estimating age in dental samples from various populations. This new version, called FIDBv3, is based exclusively on root dentin translucency and incorporates a logarithmic transformation of the variables to minimize variations caused by the nonlinear progression of translucency with advancing age. When applied to the Peruvian population, the smallest errors were recorded in the 40–49 and 50–59 age groups, with mean absolute errors between 5.73 and 6.16 years. The remaining groups showed errors of less than 11 years. Overall, age was overestimated in younger individuals (30–49 years) and underestimated in older adults (50–80 years), with no differences between the sexes.

By contrast, Becerra and Cortés [27] suggested that supervised machine learning techniques were more effective than traditional methods for estimating age in a Colombian sample. Using the measurements employed in the Lamendin method [3], they developed supervised machine learning models for age-at-death estimation. The highest accuracy was achieved using the Gaussian Process Regression (GPR) technique, which yielded a root mean square error of 3.37 years, in contrast to the 15.52 years obtained with the Lamendin method when applied to the same population. However, these findings should be interpreted with caution due to the high risk of bias identified in the methodological assessment of the study (Table 1).

De Angelis et al. [20] applied the methods of Lamendin [3] and Prince and Ubelaker [11] to estimate the age of Italian individuals who had been buried for 16 years. The difference between estimated and actual ages was statistically significant for both methods across all age ranges. Except in the 40–49 age group (which showed mean errors lower than 5 years), greater error was observed in the buried remains when compared to the results obtained in a control group used in a previous publication [29]. With the Lamendin method, the error in the age estimation of individuals over 50 years of age ranged from 10.7 to 36.8 years, compared to previously reported values of 7.3 to 18.9 years. In contrast, the Prince and Ubelaker method showed errors of between 9.5 and 35.7 years compared to 5.2 to 32.6 years in the control group. These results suggest caution when the Lamendin method is applied to forensic cases involving buried human remains, even those buried for a short period of time.

Also using the Lamendin [3] and Prince and Ubelaker [11] methods, Zorba et al. [26] estimated the age of a sample from the Greek population. Both methods showed an overestimation of age in individuals under 40 years and an underestimation in those over that age, with mean absolute errors of less than 10 years in the intermediate age group (30–59 years). The Lamendin method proved to be more accurate for the 20–39 age group, while Prince and Ubelaker’s method was more accurate for individuals over 40 years of age.

The accuracies of the Lamendin [3], Prince and Ubelaker [11], Fialho [30], and modified Fialho [30] methods were evaluated by Rocha et al. [25] in a Portuguese population. The modified Fialho method, which focuses solely on root translucency, showed the highest accuracy, followed by the Fialho, Lamendin, and Prince and Ubelaker methods. The Lamendin and Prince and Ubelaker methods underestimated age in all age groups, while the Fialho and modified Fialho methods underestimated age in females. In males, the Fialho and modified Fialho methods overestimated age in younger groups and underestimated age in the remaining groups.

Peralta et al. [23] evaluated a Peruvian sample of adult male cadavers to estimate age using different methods: Prince and Ubelaker [11], Ubelaker and Parra [31], and Vilcapoma [32]. The mean difference between the chronological and estimated ages was 3.56 years for the Prince and Ubelaker method (for white men), 2.18 years for Ubelaker and Parra, and 0.15 years for the Vilcapoma method. The results by age group indicated that the methods tended to overestimate age at younger chronological ages but to underestimate age at older chronological ages. For the 30–39 and 55–59 age groups, the Ubelaker and Parra method showed greater accuracy, while the Vilcapoma method was the most accurate for the 40–54 age group.

Finally, Quispe [24] evaluated the accuracy of the method developed by Ubelaker and Parra [31] by applying their formula to a Peruvian sample. The differences between actual and estimated ages were not statistically significant in individuals aged 30 to 49 (with mean absolute errors lower than 10 years). However, significant differences were observed in individuals over 50 years of age.

## 4. Discussion

Since the first approaches by Gustafson [8] and Bang and Ramm [10], the Lamendin method [3] has become a widely used tool in the forensic field for age-at-death estimation, undergoing various methodological modifications to improve its performance. In recent years, several issues have attracted the attention of researchers, including the accuracy of the method in different populations, the variation of the error among different age cohorts, the effect of the postmortem interval, and the influence of sex. Accordingly, the evidence identified in this systematic review is critically discussed in relation to the following key aspects: age-related estimation bias, Bayesian approaches, logarithmic modeling, population-specific equations, machine learning applications, sex-related effects, postmortem influences on root translucency, and practical implementation.

The Lamendin method [3] was evaluated by most of the studies included in this review [14,15,19,20,21,22,25,26,27]. The results by age group showed a tendency for the method to overestimate age in young adults and underestimate it in older individuals, with greater accuracy in middle-aged groups (30–60 years) [14,15,19,20,21,22,26]. This trend was also observed with the Bang and Ramm [18], Prince and Ubelaker [20,23,26], Ubelaker and Parra [23,24], and Vilcapoma [23] methods. Zorba et al. [26], in a Greek population sample, found that the Prince and Ubelaker method yielded better results for people over 40, while the Lamendin method performed better in the 20–39 age group. In a previous study by González et al. [33] in Spanish and Colombian populations, a lower mean error was found with the Prince and Ubelaker method (both the original and revised formulas) for older and younger individuals, with the Lamendin method proving more accurate in the intermediate age group.

The tendency of root translucency-based methods to overestimate age in younger adults and underestimate it in older individuals may reflect the non-linear nature of the biological process underlying dentinal translucency [4,10,13]. Root dentin translucency is associated with the progressive mineralization and occlusion of dentinal tubules. This process begins in the apical region and progressively extends towards the cervical region with advancing age, as mineral deposits within the dentinal tubules alter the optical properties of the dentin [10]. Generally, it becomes apparent during the third decade of life, although some studies have reported root dentin translucency in individuals aged 18 years [13]. In younger adults, therefore, measurable translucency may be present despite a relatively low chronological age. Moreover, interindividual variation in the extent of dentinal sclerosis may result in greater translucency than expected for a given age, potentially leading to age overestimation. Previous studies have suggested that pulpal and periodontal conditions may contribute to increased dentinal sclerosis and translucency at younger ages [34,35]. Conversely, with advancing age, the progressive mineralization of dentinal tubules may eventually lead to extensive occlusion, thereby limiting or preventing any further increase in root translucency [10,34,35]. In addition, the substantially greater amount of dentin in the cervical region of the root compared to the apical portion may partly explain the slower development of root translucency at older ages, as the deposition of inorganic salts probably requires more time to produce detectable changes [13]. Odontoblast activity may also contribute to this phenomenon, given their active role in mineral deposition and their age-related functional decline [14]. Therefore, the ability of root translucency to discriminate between age groups may decrease at advanced ages [14,15,19,20,21,22,23,24,25,26]. This phenomenon, known as the “trajectory effect” [36], reflects the increasing variability of age-related biological changes with advancing age, resulting in broader error intervals and reduced accuracy of age estimation [7,11,13,20,21,26,31,33,37,38].

Nonetheless, the inaccurate age estimation results have also been partially attributed to statistical issues [37,39]. Although the impact of the trajectory effect cannot be eliminated [13,14,38], it can be mitigated by using Bayesian statistics in the analysis [13,14,15,37,40]. Bayesian approaches may reduce some age-related estimation bias and provide a more informative representation of uncertainty; however, the underlying biological limitations remain. Similarly, logarithmic regressions yielded better results than traditional linear regression, particularly in adults over 65–70 years [14,22]. Building on these findings, the incorporation of logarithmic transformations into Bayesian models has contributed to improving variance homogeneity and mitigating the trajectory effect [14,15]. Beyond their potential to improve accuracy, Bayesian methods offer important conceptual advantages by explicitly incorporating uncertainty into age estimation. Bayesian approaches allow age estimates to be expressed probabilistically, providing a distribution of plausible ages while integrating prior information with the observed evidence [13]. This probabilistic framework may be particularly valuable in forensic contexts, where uncertainty is inherent in age estimation and should be considered when interpreting individual age estimates.

The FIDB [13] and its subsequent versions [14,15] represent applications of the Bayesian framework to forensic age estimation. These approaches combine dental markers with reference data from multiple populations to generate probabilistic age estimates. In this context, the FIDBv2 [14] was proposed as a potential alternative when no additional data on the individual’s population of origin is available or no previous population-specific studies have been conducted. The average difference between actual and estimated ages found by Garizoain et al. [14] in Greek and Colombian populations was less than 10 years in individuals aged 30 to 60, similar to that reported by Garizoain et al. [38] using the first version of the FIDB [13]. Parra et al. [15] found that using the FIDBv3 algorithm and root translucency assessment as the sole parameter resulted in errors of around 6 years in the Peruvian population in the 40–59 age cohort, suggesting the validity of this type of analysis across different populations. Rocha et al. [25] also suggested that simplified methods excluding periodontal recession may have potential for age estimation. However, these findings should be interpreted with caution, as model performance may also be influenced by methodological factors such as recalibration, statistical transformation, sample composition, and validation procedures. Therefore, additional studies with larger and more diverse populations are needed to assess the applicability and generalizability of these approaches across different forensic contexts.

The development of population-specific formulas has been suggested as a valuable approach in dental age estimation, as these models appear to provide better results than the original formulas [18,19,23,25,30,31,32]. Alvarado and Requena [18] reported that the population-specific formula for a Peruvian sample exhibited slightly lower errors than the original formula developed by Bang and Ramm [10]. Baz et al. [19], using the Lamendin method [3], also developed a specific formula for the study population (Brazilian), achieving better performance in individuals aged 60 to 89 years. Peralta et al. [23] determined that the Vilcapoma method [32] and the Ubelaker and Parra method [31], both adapted to the Peruvian population, were more accurate than the Prince and Ubelaker method [11] when applied to the study sample (Peruvian). These findings partially contrast with those of Vilcapoma [32], who found that the Prince and Ubelaker method [11] was more accurate than that of Ubelaker and Parra [31]. Rocha et al. [25] demonstrated better accuracy with the Fialho and modified Fialho methods [30], which adapted the original formula developed by Lamendin et al. [3] to a Portuguese population. The apparently superior performance of population-specific formulas should be interpreted cautiously, as it may partly reflect optimization to the development sample rather than genuine biological differences between populations [19]. This may lead to overfitting, whereby model performance is overestimated when tested in similar samples to those used for model development. Independent external validation is therefore essential to determine whether the reported performance is reproducible in other samples from the same target population. The added value of population-specific formulas should be carefully evaluated, particularly when the available evidence suggests that population variation may have a limited impact on age estimation [11,13,31,33].

Machine learning techniques are emerging as promising tools in forensic age estimation, particularly in image and biometric data analysis, with the potential to improve accuracy and efficiency [41]. However, their performance requires further validation in larger and independent samples before routine forensic application. Using the GPR technique, Becerra and Cortés [27] significantly reduced the error in estimates made using the Lamendin method. Nevertheless, this finding warrants cautious interpretation, as the model may have been influenced by the characteristics of the development sample, particularly its size and composition, raising the possibility of overfitting and limiting its generalizability. The high risk of bias identified in this study, mainly due to insufficient information or uncertainties about the inclusion criteria, description of the setting, handling of confounders, and outcome measures (Table 1), further supports the need for independent external validation before these approaches can be routinely applied. Moreover, the limited interpretability of some machine learning models and the need for transparent and scientifically defensible age estimates remain important challenges to their implementation in forensic practice.

Regarding sex, Garizoain et al. [21] and Parra et al. [15], in line with other authors [3,13,38,42], found no influence of this variable on age-at-death estimation. Conversely, Rocha et al. [25] observed sex differences in the estimates, as previously reported by others [11]. However, these differences may at least be partly due to the sample size and sex distribution within the age groups [21,22,25].

De Angelis et al. [20] showed that caution should be exercised when the Lamendin method [3] is applied to human remains buried for several years, because burial in soil can alter root translucency. In this regard, several authors have indicated that postmortem factors can affect the applicability of the Lamendin method to archaeological and historical samples [7,37,43]. González et al. [33] found that the Prince and Ubelaker method was equally accurate in teeth from living subjects and those from recent skeletal remains, whereas the Lamendin method was less accurate in the skeletal remains. The studies included in this review measured root dental translucency in teeth from adult corpses obtained from autopsies, cemeteries, and osteological collections, with a date of death no earlier than the mid-20th century. The results were comparable to those of studies that also evaluated teeth from living subjects [13,38,44,45]. Therefore, the Lamendin method appears to be useful when applied to teeth from recently deceased individuals (within several decades), as previously reported [13]. However, its usefulness may be limited in cases of longer postmortem intervals in which diagenesis has affected the remains [13]. For further improvement, a detailed comparison between the research of cadaveric teeth and freshly extracted teeth must be considered.

Beyond accuracy, the forensic applicability of these methods should be considered in terms of their practical implementation. Factors such as ease of use, repeatability, the applicability of the method to isolated teeth, preservation requirements, and the specific forensic context may influence their suitability for casework. Methods that are simple, reproducible, and applicable to isolated or well-preserved teeth may offer practical advantages, whereas approaches requiring specific imaging procedures, extensive sample preparation, or specialized equipment may be more difficult to implement routinely. Conventional approaches [18,19,20,21,22,23,24,25,26,28] generally involve direct assessment and measurement of root dentin translucency and may be relatively straightforward to apply when the required dental structures are adequately preserved, including in cases involving isolated teeth. In contrast, approaches incorporating supervised machine learning techniques may require digital measurements and computational procedures, potentially involving additional technical resources [27]. The FIDB and its subsequent versions [14,15] provide probabilistic age estimates within a Bayesian framework and are publicly available through an online platform [46], which may facilitate their implementation in forensic and research settings. Therefore, the selection of an age-estimation method in forensic practice should consider not only its reported accuracy but also its operational feasibility and suitability for the specific case circumstances. Nonetheless, these practical considerations should also be interpreted in light of the methodological limitations and risk of bias identified in the included studies, as previously discussed, which may further affect the generalizability and forensic applicability of the reviewed methods.

Several limitations should be acknowledged when interpreting the findings of this review. First, the literature search was restricted to studies published between 2015 and 2025 and to publications available in English and Spanish, which may have limited the inclusion of potentially relevant evidence. Second, the included studies showed considerable methodological heterogeneity, with variations in tooth types (incisors, premolars, and/or canines), sample sources (autopsies, cemeteries, and/or osteological collections), measurement protocols, and evaluated age groups, which limited direct comparison of their findings. These methodological differences may influence the detection and measurement of root dentin translucency and related variables and, consequently, the reported accuracy of age estimates. In addition, the inconsistent reporting of accuracy measures limited the possibility of comparing the performance of different methods. Therefore, greater standardization of tooth selection, specimen preparation, and root dentin translucency assessment and measurement procedures may be necessary to improve the reproducibility and comparability of age estimation methods. Third, two included studies were classified as having a high risk of bias, which should be considered when interpreting the overall findings. Finally, a meta-analysis could not be performed because of the substantial heterogeneity across studies, including differences in the evaluated methods, tooth types, age ranges, sample sources, statistical approaches, and reported performance measures.

## 5. Conclusions

Root dentin translucency is a useful marker for adult age-at-death estimation, although its performance varies according to age group, statistical model, sample characteristics, and postmortem preservation. The methods evaluated generally show greater accuracy in intermediate age groups (mean errors lower than 10 years), whereas age tends to be overestimated in young adults and underestimated in older individuals. The error in estimates increases with age and may be reduced using Bayesian statistics in the analyses. Similarly, logarithmic regressions have shown better results than traditional linear regression in adults over 65 years of age. Recent translucency-only models have shown promising performance, but their apparent superiority over multi-parameter methods has not yet been established. International forensic dental databases may offer a useful alternative when population-specific reference data are unavailable, although continued external validation remains necessary. Overall, the current evidence remains limited by the relatively small number of studies and substantial methodological heterogeneity. Further research using larger and more diverse samples, standardized methodologies, and independent external validation is needed to establish the generalizability and forensic applicability of these approaches across different populations and forensic contexts.

## Data Availability

No new data were created or analyzed in this study. Data sharing is not applicable to this article.

## Supplementary Materials

The following supporting information can be downloaded at https://www.mdpi.com/article/10.3390/diagnostics16183055/s1, File S1: Prisma 2020 Checklist; File S2: Search strategies.

## Abbreviations

The following abbreviations are used in this manuscript:

PECO	Population, Exposure, Comparison, and Outcome
PRISMA	Preferred Reporting Items for Systematic Reviews and Meta-Analyses
PROSPERO	International Prospective Register of Systematic Reviews
JBI	Joanna Briggs Institute
FIDB	Forensic International Dental Database
GPR	Gaussian Process Regression
