# Supplementary material for: Root Translucency for Age-at-Death Estimation in Adults: A Systematic Review
Source: Diagnostics (Basel). 2026 Sep 20;16(18):3055. doi: 10.3390/diagnostics16183055 (PMC13605641; doi:10.3390/diagnostics16183055)
Supplement: Supplementary file 1 [file diagnostics-16-03055-s001.zip › Supplementary_File_S2.pdf]

Supplementary File S2. Search strategies

Database	Search strategy
**PubMed/MEDLINE, Scopus, Web of Science, SciELO**	root translucency OR root transparency AND age estimation<br>Publication date: 2015-2025
	Lamendin method AND age-at-death<br>Publication date: 2015-2025
	Prince and Ubelaker method AND age-at-death<br>Publication date: 2015-2025
**Google Scholar**	**Exact phrase:** "age estimation" + **At least one of these words:** "root"; "translucency"; "transparency" + **Where my words occur:** Article title + **Show articles dated between:** 2015-2025
	**All of these words:** "age"; "at"; "death" + **Exact phrase:** "Lamendin method" + **Where my words occur:** Anywhere in the article + **Show articles dated between:** 2015-2025
	**All of these words:** "age"; "at"; "death" + **Exact phrase:** "Prince and Ubelaker method" + **Where my words occur:** Anywhere in the article + **Show articles dated between:** 2015-2025
